# PKMiner: a database for exploring type II polyketide synthases

**DOI:** 10.1186/1471-2180-12-169

**Published:** 2012-08-08

**Authors:** Jinki Kim, Gwan-Su Yi

**Affiliations:** 1 Department of Information and Communications Engineering, KAIST, Daejeon, 305-701, South Korea; 2 Department of Bio and Brain Engineering, KAIST, Daejeon, 305-701, South Korea

## Abstract

**Background:**

Bacterial aromatic polyketides are a pharmacologically important group of natural products synthesized by type II polyketide synthases (type II PKSs) in actinobacteria. Isolation of novel aromatic polyketides from microbial sources is currently impeded because of the lack of knowledge about prolific taxa for polyketide synthesis and the difficulties in finding and optimizing target microorganisms. Comprehensive analysis of type II PKSs and the prediction of possible polyketide chemotypes in various actinobacterial genomes will thus enable the discovery or synthesis of novel polyketides in the most plausible microorganisms.

**Description:**

We performed a comprehensive computational analysis of type II PKSs and their gene clusters in actinobacterial genomes. By identifying type II PKS subclasses from the sequence analysis of 280 known type II PKSs, we developed highly accurate domain classifiers for these subclasses and derived prediction rules for aromatic polyketide chemotypes generated by different combinations of type II PKS domains. Using 319 available actinobacterial genomes, we predicted 231 type II PKSs from 40 PKS gene clusters in 25 actinobacterial genomes, and polyketide chemotypes corresponding to 22 novel PKS gene clusters in 16 genomes. These results showed that the microorganisms capable of producing aromatic polyketides are specifically distributed within a certain suborder of *Actinomycetales* such as *Catenulisporineae, Frankineae, Micrococcineae, Micromonosporineae, Pseudonocardineae, Streptomycineae, and Streptosporangineae*.

**Conclusions:**

We could identify the novel candidates of type II PKS gene clusters and their polyketide chemotypes in actinobacterial genomes by comprehensive analysis of type II PKSs and prediction of aromatic polyketides. The genome analysis results indicated that the specific suborders in actinomycetes could be used as prolific taxa for polyketide synthesis. The chemotype-prediction rules with the suggested type II PKS modules derived using this resource can be used further for microbial engineering to produce various aromatic polyketides. All these resources, together with the results of the analysis, are organized into an easy-to-use database PKMiner, which is accessible at the following URL: http://pks.kaist.ac.kr/pkminer. We believe that this web-based tool would be useful for research in the discovery of novel bacterial aromatic polyketides.

## Background

Polyketides are a large family of secondary metabolites with diverse structures and biological activities. Many of these are clinically important compounds with antibiotic, antifungal, and anticancer properties [1]. Polyketide biosynthesis is catalyzed by a group of enzymes called polyketide synthases (PKSs). The carbon chain of polyketides is formed through stepwise decarboxylative condensation of acyl-thioester units by a coordinated group of PKS domains. The genes encoding PKS are usually clustered with their auxiliary and regulatory elements on the genome, and their products are classified into types I, II, and III depending on their domain organization [2].

**Figure 1:**
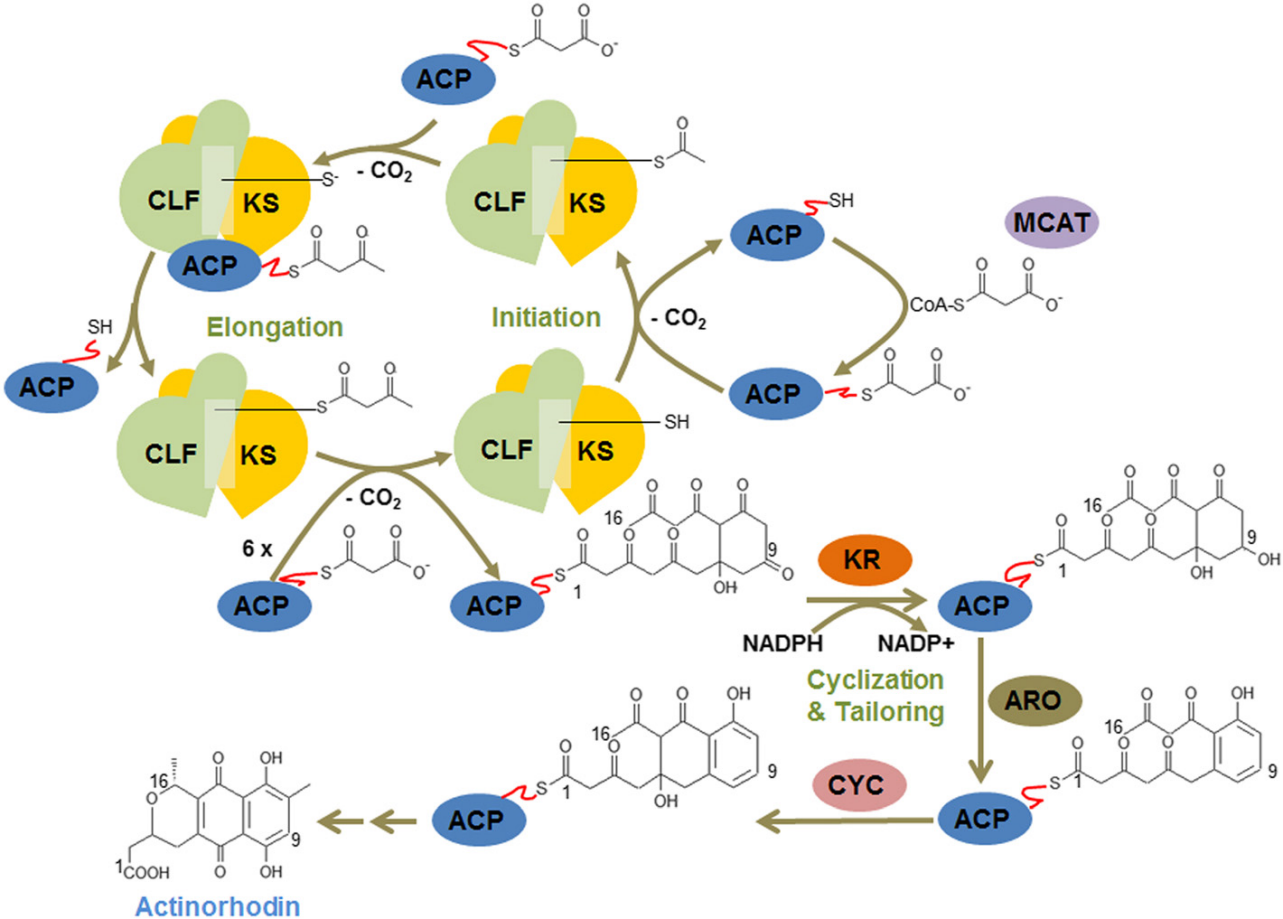
**Schematic diagram depicting the activity of type II PKS domains with actinorhodin biosynthesis as an example.** Heterodimeric KS and CLF domains catalyze chain initiation and elongation through decarboxylative condensation of malonyl building blocks, an ACP domain delivers malonyl building blocks to the KS-CLF, and a MCAT domain supplies malonyl groups to the ACP domain. The collective action of these type II PKS domains lead to the formation of highly reactive poly-β-keto intermediates. This nascent polyketide chain is modified into a specific folding pattern by tailoring enzyme domains such as those of KR, ARO, and CYC. The KR domain reduces carbonyl group at a specific position of the polyketide chain, and the ARO and CYC domains control chain folding by catalyzing one or more regiospecific cyclization in the polyketide chain. Whereafter polyketide chain is modified by various tailoring enzymes into actinorhodin.

Bacterial aromatic polyketides such as tetracyclines and actinorhodin are polycyclic phenolic compounds that are assembled by type II PKSs. A characteristic of type II PKSs is domain composition with a maximum of 2 domains in each type II PKS and the iterative use of domains to synthesize a polyketide product [3]. Figure 1 shows the schematic diagram depicting the activity of type II PKS domains with actinorhodin biosynthesis as an example. Heterodimeric ketosynthase (KS) and chain length factor (CLF) domains catalyze chain initiation and elongation through decarboxylative condensation of malonyl building blocks, an acyl carrier protein (ACP) domain delivers malonyl building blocks to the KS-CLF, and a malonyl-CoA: ACP transacylase (MCAT) domain supplies malonyl groups to the ACP domain. The collective action of these type II PKS domains lead to the formation of highly reactive poly-β-keto intermediates. This nascent polyketide chain is modified into a specific folding pattern by tailoring enzyme domains, such as those of ketoreductase (KR), aromatase (ARO), and cyclase (CYC). The KR domain reduces carbonyl groups at a specific position of the polyketide chain, and the ARO and CYC domains control chain folding by catalyzing one or more regiospecific cyclization in the polyketide chain. Typical primary products of these type II PKSs are polyphenols that can be classified into 7 polyketide chemotypes: linear tetracyclines, anthracyclines, benzoisochromanequinones, tetracenomycins, aureolic acids, and angular angucyclines, as well as a group of pentagular polyphenols [4]. Additional modification by several elaborate tailoring enzymes such as dimerases, P450 monooxygenases, methyltransferases, and glycosyltransferases can further diversify phenolic polycyclic compounds such as actinorhodin [5].

Currently, a vast majority of polyketides is derived from a single *Actinomycetes* genus, *Streptomyces*[6]. It is difficult to culture most microorganisms on earth that produce aromatic polyketides, under standard laboratory conditions because of their different growth rates and difficulties in laboratory manipulation [7]; this evidences the fact that there are a few aromatic polyketide producers and that the complete realm of these microorganisms remains to be explored. Furthermore, studies on type II PKSs and their polyketides have been performed on a limited number of genomes. However, the current progress of computational methods and substantial increase of genome sequencing data has created new possibilities to comprehensively characterize polyketide-producing genomes and increase the number of valuable resources in this field [8].

In order to discover novel aromatic polyketides based on genome mining, it is essential to comprehensively analyze various type II PKSs in different organisms to detect type II PKSs and analyze the correlation between domain organizations and polyketide structures. A number of experimental studies have explained in detail the biosynthetic functions of type II PKSs domains, which are encoded by a type II PKS gene cluster and have polyketide products, and most of the identified type II PKS domains are known to be conserved in related species; therefore, it is possible to discover new type II PKS domains on the basis of sequence similarities in homologous type II PKS domains. Furthermore, the comprehensive phylogenetic analysis of tailoring enzymes such as ARO and CYC provides details about their biosynthetic function in regulation of the metabolic pathway determining aromatic polyketide chemotypes [4]. This finding allows us to investigate the possibility of analyzing type II PKS domain compositions in type II PKS gene clusters with respect to aromatic polyketide chemotypes. Currently, there are several sequence-based polyketide gene cluster analysis systems for type I and type III PKSs, such as NRPS-PKS, ASMPKS, ClustScan, NP. Searcher, and antiSMASH [9-13]. Among these, antiSMASH is the only system that supports the analysis of type II PKS gene cluster. This system identifies gene clusters of type II PKS-specific domains such as KS, CLF, and ARO by using sequence-based classification. However, it is difficult to identify other type II PKSs and associate the gene cluster with the chemical structure of type II PKS products.

Here, we performed a comprehensive computational analysis of type II PKSs and their gene clusters in actinobacterial genomes. First, we carried out an exhaustive sequence analysis of known type II PKSs by using homology-based sequence clustering for the identification of type II PKS subclasses. This analysis enabled us to develop type II PKS domain classifiers and derive polyketide chemotype-prediction rules for the analysis of type II PKS gene cluster. Using these rules, we analyzed available actinobacterial genomes and predicted novel type II PKSs and PKS gene clusters together with potential bacterial aromatic polyketide chemotypes. The predicted type II PKS gene clusters were verified by using information from the available literature. All the resources, together with the results of the analysis, are organized into an easy-to-use database PKMiner, which is accessible at http://pks.kaist.ac.kr/pkminer.

## Construction and content

### Data sources

A total of 42 type II PKS gene clusters having type II PKS proteins were identified from individual literature and their sequence information was collected from the National Center for Biotechnology Information (NCBI) nucleotide database. A total of 37 bacterial aromatic polyketide chemotypes corresponding to type II PKS gene clusters were collected from literature and the NCBI pubchem database (see Additional file 1: Table S1).

To fully download completely sequenced genomes from the NCBI genome database, we made custom perl script using the NCBI E-utils based on actinobacteria taxonomy. As a result, we collected a total of 319 actinobacterial genome sequences. (see Additional file 1: Table S2).

### Type II PKS identification

We identified a total of 280 known type II PKS proteins with functional activity from 42 aromatic polyketide gene clusters (see Additional file 1: Table S3). They include type II PKS classes such as keto synthase (KS), chain length factor (CLF), acyl carrier protein (ACP), keto reductase (KR), aromatase (ARO), cyclase (CYC), keto synthase III (KSIII), acyl CoA ligase (AL), acyl transferase (AT), malonyl-CoA: ACP transacylase (MCAT), and thioesterase (TE). We performed homology based clustering analysis for the sequences of each type II PKS class based on sequence similarity and biosynthetic function because several classes of type II PKSs such as KR, ARO and CYC have various different types of subclasses [4,14] and the Pfam search tool [15] and the Conserved Domain Database (CDD) server of NCBI [16] often failed to identify domains in type II PKS protein sequences (see Additional file 1: Table S3). The sequences of each type II PKS class were grouped into clusters using the BLASTCLUST from the BLAST software package [17]. The number of cluster is determined when type II PKSs with different biosynthetic function were accurately separated. The subclasses determined by the sequence clustering analysis matched well with the known functional subclasses reported in literature for KR, ARO, and CYC. There was no evidence showing separate functional groups in KS III class yet but our analysis showed that the sequence-based subclasses of KS III have discriminating patterns as significant as the subclasses of other PKS domains. We maintain these subclasses of KS III as the potential subgroups of KS III in our study. We could confirm that the pattern of sequence conservation in C7 KR cluster is different from that of C9 KR cluster. We also could confirm that ARO clusters agreed well with previously known subgroups such as a monodomain and two didomain types. The N-terminal and C-terminal domain types of didomain aromatase and monodomain types of aromatases from literature are mapped to ARO subclasses a, b, and c, respectively [18]. In addition, CYC clusters well correspond to previously reported phylogenetic analysis result of type II PKS tailoring enzymes, which shows that the ring topology of aromatic polyketide correlates well with the types of cyclases [4]. As a result, we identified that 11 type II PKS classes were clustered into a total of 20 types of subclasses with distinct biosynthetic function and different average length of domain sequences as shown in Table 1 (see Additional file 1: Table S4).

**Table 1:** Result of homology based clustering analysis for 280 known type II PKSs

**Domain**	**Subfamily**	**Biosynthetic function**	**Number of domains in each subfamily**	**Total number of domains**	**Average length**
KS	a	Keto synthase	43	43	416
CLF	a	Chain length factor	43	43	407
ACP	a	Acyl carrier protein	44	44	78
KR	a	C9 ketoredution	25	30	214
	b	C7 ketoredution	5		204
ARO	a	First and second ring cyclization in reduced chain of Ben, Ang, Tet and Ant	28	67	132
	b	First and second ring cyclization in reduced chain of Ben, Ang, Tet and Ant	28		143
	c	First and second ring cyclization in unreduced chain of Pen and Tet	11		134
CYC	a	Second and third ring cyclization in Pen and Tet	11	57	93
	b	Second and third ring cyclization in Aur and Ant	10		235
	c	Final cyclization in Ang, Pen and Tet	19		102
	d	Final cyclization in Ben	6		176
	e	Final cyclization in Aur	5		121
	f	Final cyclization in Ant	6		127
KSIII	a	Keto synthase III	4	7	218
	b	Keto synthase III	3		231
AL	a	Acyl-CoA ligase	3	3	389
AT	a	Acyl transferase	10	10	320
MCAT	a	Malonyl-CoA: ACP transferase	3	3	309
TE	a	Thioesterase	1	1	232

Unc-unclassified, Ang-Angucyclines, Ant-Anthracyclines, Ben- Benzoisochromanequinones, Pen- Pentangular polyphenols, Tet- Tetracenomycins, Aur- Tetracyclines/aureolic acids.

For each of type II PKS domain, this table shows the subfamily, biosynthetic function, number of domains in each subfamily, total number of domains and the average length present in 280 known type II PKSs.

### Construction of type II PKS domain classifiers

Type II PKS domain classifiers were developed for each type II PKS subclass using combination of hidden Markov Model (HMM) and sequence pairwise alignment based support vector machine (SVM) [19]. The profiled HMM of each type II PKS domain was trained with the sequences of the corresponding domain. HMM calculation was performed using the HMMER software package [20]. For the construction of SVM classifiers, we used the available software package libSVM [21] to implement SVM on our training datasets. The feature vector for SVM classifier was generated from the scores of pairwise sequence comparison by Smith-Waterman algorithm implemented in SSEARCH from the FASTA software package [22]. The SVM model of each domain subfamily was trained with the sequences of the training dataset. We performed training testing cycles using in-house PERL scripts. We used RBF kernel to train and test our SVM models. The parameter value C and r of kernel function were optimized on the training datasets by cross-validation. The best parameter set was determined when the product of sensitivity and specificity maximize the prediction accuracy. To evaluate the performance of each domain classifier, the following predictive performance measures were used: Sensitivity (SN) = TP/(TP + FN), Specificity (SP) = TN/(TN + FP), Accuracy (AC) = (TP + TN)/(TP + FP + TN + FN) and Matthews correlation coefficient (MCC) = {(TP x TN) – (FN x FP)}/√(TP + FN) x (TN + FP) x (TP + FP) x (TN + FN) where TP, TN, FP and FN are true positive, true negative, false positive and false negative predictions, respectively. We took type II PKS domain subfamily sequences as the positive set and randomly selected sequences from non-type II PKS domains as the negative set. Depending on the dataset size, 4-fold cross-validation (n ≥ 20) or leave-one-out cross-validation (n < 20) were applied. The average of 10 repeated cross-validation results were used to calculate the performances.

Table 2 shows the results of evaluation of type II PKS domain classifiers. As shown in Table 2, the two types of domain classifiers show different domain classification accuracy. Among 15 type II PKS domain subfamilies, domain classifiers based on SVM outperformed that based on HMM for 12 type II PKS domain subfamilies. It indicates that classification performance of type II PKS domain could vary depending on the type of domain classifier. These domain classifiers remarkably show high classification accuracy. For 10 domain subfamilies, each domain classifier showing the higher performance reaches 100 % in classification accuracy. Therefore, we finally obtained high performance domain classifiers composed of profiled HMM and sequence pairwise alignment based SVM.

**Table 2:** Evaluation of type II PKS domain classifiers using profiled HMM and sequence pairwise alignment based SVM with 4- fold cross-validation (n > 20) and leave-one-out cross-validation (n < 20)

**Domain**	**Subfamily**	***n***	**HMM**				**SVM**			
			**SN (%)**	**SP (%)**	**AC (%)**	**MCC (%)**	**SN (%)**	**SP (%)**	**AC (%)**	**MCC (%)**
KS	a	43	100	100	100	100	100	100	100	100
CLF	a	43	100	100	100	100	100	100	100	100
ACP	a	44	100	97.78	98.86	97.75	93.26	97.38	95.23	90.55
KR	a	25	100	100	100	100	100	100	100	100
	b	5	100	100	100	100	100	100	100	100
ARO	a	29	98.98	100	99.48	98.97	100	93.85	96.72	93.65
	b	29	96.67	90.38	93.3	86.62	100	100	100	100
	c	11	96.67	89.74	93.06	86.41	100	91.67	95.45	91.29
CYC	a	19	92.97	84.11	88.03	76.57	100	100	100	100
	b	11	92.97	79.52	85	71.24	100	91.67	95.45	91.29
	c	10	76.7	94.5	83.38	68.95	100	100	100	100
	d	6	93.75	80.45	85.91	73	100	100	100	100
	e	5	77.53	96.29	84.53	71.4	100	100	100	100
	f	6	100	100	100	100	100	75	83.33	70.71
AT	a	10	77.76	95.77	84.56	71.28	83.33	100	90	81.65

SN-sensitivity, SP-Specificity, AC-Accuracy, MCC-Matthews correlation coefficient.

### Derivation of prediction rules for aromatic polyketide chemotype

Since type II PKS subclasses can be identified correctly by clustering the sequence of type II PKS proteins, we attempted to identify correlation between type II PKS domain organization and aromatic polyketide chemotype. Previous study has suggested that the ring topology of aromatic polyketide correlates well with the types of cyclases [4]. We therefore examined domain combinations of type II PKS ARO and CYC by mapping these domain subfamilies onto aromatic polyketide chemotypes (see Additional file 1: Table S5)

Table 3 shows the results of the type II PKS ARO and CYC domain combinations corresponding to each aromatic polyketide chemotype. These results reveal that there are unique and overlapped domain combinations for six aromatic polyketide chemotypes. While angucyclines, anthracyclines, benzoisochromanequinones and pentangular polyphenols chemotypes have 7 unique ARO and CYC domain combinations, there are two pairs of overlapped ARO and CYC domain combinations between anthracyclines and tetracyclines/aureolic acids chemotypes and between pentangular polyphenols and tetracenomycins chemotypes. We thus attempted to derive aromatic polyketide chemotype-prediction rules based on the above results. However, the above results also show overlapped domain combinations between aromatic polyketide chemotypes, preventing accurate prediction of aromatic polyektide chemotype. We therefore integrated domain combinations with sequence homology for the prediction of aromatic polyketide chemotype, which is inspired from previous study showing that homologous type II PKS tailoring enzymes such as ARO and CYC tend to be clustered in the same clade of phylogenetic tree [4]. The aromatic polyketide chemotype classification rules based on domain combinations and sequence homology are as follows: 1) for type II PKS gene cluster mapped onto aromatic polyketide chemotype with unique domain combination, we assigned corresponding polyketide chemotype into type II PKS gene cluster. 2) for type II PKS gene cluster mapped onto aromatic polyketide chemotype with overlapped domain combination, we assigned the most abundant polyketide chemotype of homologs of ARO and CYC onto the type II PKS gene cluster.

**Table 3:** Type II PKS ARO/CYC domain combinations of aromatic polyketide chemotype

**Polyketide Chemotype**	**Type II PKS domain subfamilies**									**Uniqueness**
	**ARO_a**	**ARO_b**	**ARO_c**	**CYC_a**	**CYC_b**	**CYC_c**	**CYC_d**	**CYC_e**	**CYC_f**	
Angucyclines	√	√		√						√
Anthracyclines	√	√				√			√	√
	√	√							√	√
	√	√				√		√		x
	√					√				√
Benzoisochromanequinones	√	√					√			√
		√					√			√
Pentangular polyphenols			√		√					√
			√	√	√					x
Tetracenomycins			√	√	√					x
Tetracyclines/aureolic acids	√	√				√		√		x

For each aromatic polyketide chemotype, this table shows ARO/CYC domain combinations of type II PKS gene clusters. The uniqueness column indicates whether or not type II PKS ARO/CYC domain combinations overlap between aromatic polyketide chemotypes.

### Predicted type II PKS and aromatic polyketide chemotypes in actinobacterial genomes

319 currently available actinobacterial genomes were analyzed using type II PKS domain classifiers and aromatic polyketide chemotype-prediction rules. For the discovery of type II PKS gene clusters in genome sequence, both upstream and downstream predicted type II PKS sequences with pairwise distance less than 15,000 base pairs in genomic location were considered as clustered type II PKS genes. The type II PKS gene clusters with type II PKS KS and CLF domain were only chosen as valid type II PKS gene cluster candidates capable of producing aromatic polyketide.

Table 4 shows 231 type II PKSs in 40 type II PKS gene clusters for 25 actinobacterial genomes (see Additional file 1: Table S6). It exhibits that among 40 type II PKS gene clusters, 36 type II PKS gene clusters are classified into one of the six aromatic polyketide chemotypes. 4 type II PKS gene clusters remains unclassified polyketide chemotype because they have incomplete type II PKS domain composition in which aromatic polyketide chemotype could not be predicted. The distribution of predicted aromatic polyketide chemotype includes 4 types of aromatic polyketide chemotype such as angucyclines, anthracyclines, benzoisochromanequinones and pentangular polyphenols. Especially, it turns out that pentangular polyphenol is the most abundant polyketide chemotype predicted by the largest number of organisms. It also revealed type II PKS members that were so far not annotated as type II PKS. These type II PKS members all have single domain and are located within the gene cluster of other type II PKSs. These include 11 proteins that were marked as hypothetical or unknown function protein and 1 protein as modular polyketide synthase. Additionally we could confirm the proposed annotation of further 3 proteins that were marked as putative type II PKS.

**Table 4:** Microorganisms with type II PKS gene clusters from the analysis of 319 actinobacterial genomes

**Genus**	**Species**	**Size (bp)**	**# of Type II PKSs**	**Polyketide Chemotype**							**Reference**
				**Unc**	**Ang**	**Ant**	**Ben**	**Pen**	**Tet**	**Aur**	
*Amycolatopsis*	*Amycolatopsis mediterranei* U32	10,236,715	6					1			[23]
*Catenulispora*	*Catenulispora acidiphila* DSM 44928	10,467,782	18		1		1	1			
*Cellulomonas*	*Cellulomonas flavigena* DSM 20109	4,123,179	4					1			
*Frankia*	*Frankia alni* str. ACN14A	7,497,934	5					1			[24]
*Frankia*	*Frankia* sp. CcI3	5,433,628	17	1			1	1			[24]
*Frankia*	*Frankia* sp. EAN1pec	8,982,042	5					1			[24]
*Frankia*	*Frankia* sp. EuI1c	8,815,781	12				1	1			
*Frankia*	*Frankia symbiont of Datisca glomerata*	5,323,186	15					3			
*Geodermatophilus*	*Geodermatophilus obscurus* DSM 43160	5,322,497	6					1			
*Micromonospora*	*Micromonospora aurantiaca* ATCC 27029	7,025,559	15			1		1			
*Micromonospora*	*Micromonospora* sp. L5	6,962,533	15			1		1			
*Nocardiopsis*	*Nocardiopsis dassonvillei* subsp. *dassonvillei* DSM 43111	5,767,958	3	1							
*Saccharomonospora*	*Saccharomonospora viridis* DSM 43017	4,308,349	6					1			
*Salinispora*	*Salinispora arenicola* CNS-205	5,786,361	6					1			[25]
*Salinispora*	*Salinispora tropica* CNB-440	5,183,331	10	1				1			[26]
*Streptomyces*	*Streptomyces avermitilis* MA-4680	9,025,608	11		1			1			[27]
*Streptomyces*	*Streptomyces coelicolor* A3(2)	8,667,507	12				1	1			[28]
*Streptomyces*	*Streptomyces rochei* plasmid pSLA2-L DNA	210,614	6				1				[29]
*Streptomyces*	*Streptomyces scabiei* 87.22	10,148,695	6					1			
*Streptomyces*	*Streptomyces sp. Sirex*AA-E	7,414,440	17		2			1			
*Streptomyces*	*Streptomyces violaceusniger* Tu 4113	10,657,107	6					1			
*Streptosporangium*	*Streptosporangium roseum* DSM 43021	10,341,314	6					1			
*Thermobifida*	*Thermobifida fusca* YX	3,642,249	7		1						
*Thermomonospora*	*Thermomonospora curvata* DSM 43183	5,639,016	7				1				
*Verrucosispora*	*Verrucosispora maris* AB-18-032	6,673,976	10	1				1			

Unc-unclassified, Ang-Angucyclines, Ant-Anthracyclines, Ben- Benzoisochromanequinones, Pen- Pentangular polyphenols, Tet- Tetracenomycins, Aur- Tetracyclines/aureolic acids.

For each microorganism with type II PKS gene cluster, this table shows genome size, number of type II PKSs, predicted aromatic polyketide chemotype and reference.

To verify the results of our analysis, we have compared the type II PKS gene cluster with available literature information. It shows that 14 type II PKS gene clusters in 9 microbial organisms were reported in literature. However, there is no description for aromatic polyketide chemotype corresponding to type II PKS gene cluster except those in *Steptomyces coelicolor* A3(2), which are already included in our known type II PKSs. It also reveals that 16 microbial organisms are not currently reported as having type II PKS gene clusters. There were 22 novel type II PKS gene clusters for which the corresponding polyketide chemotypes could be predicted.

### Database architecture

PKMiner was implemented on the relational database system MySQL. A custom-made parsers and modules in the backend were developed in Perl. The Web interface was designed and implemented using Perl and Asynchronous Javascript and XML (AJAX). AJAX was adopted for making Web pages more interactive without page reloading.

## Utility

### The browsing interface

**Figure 2:**
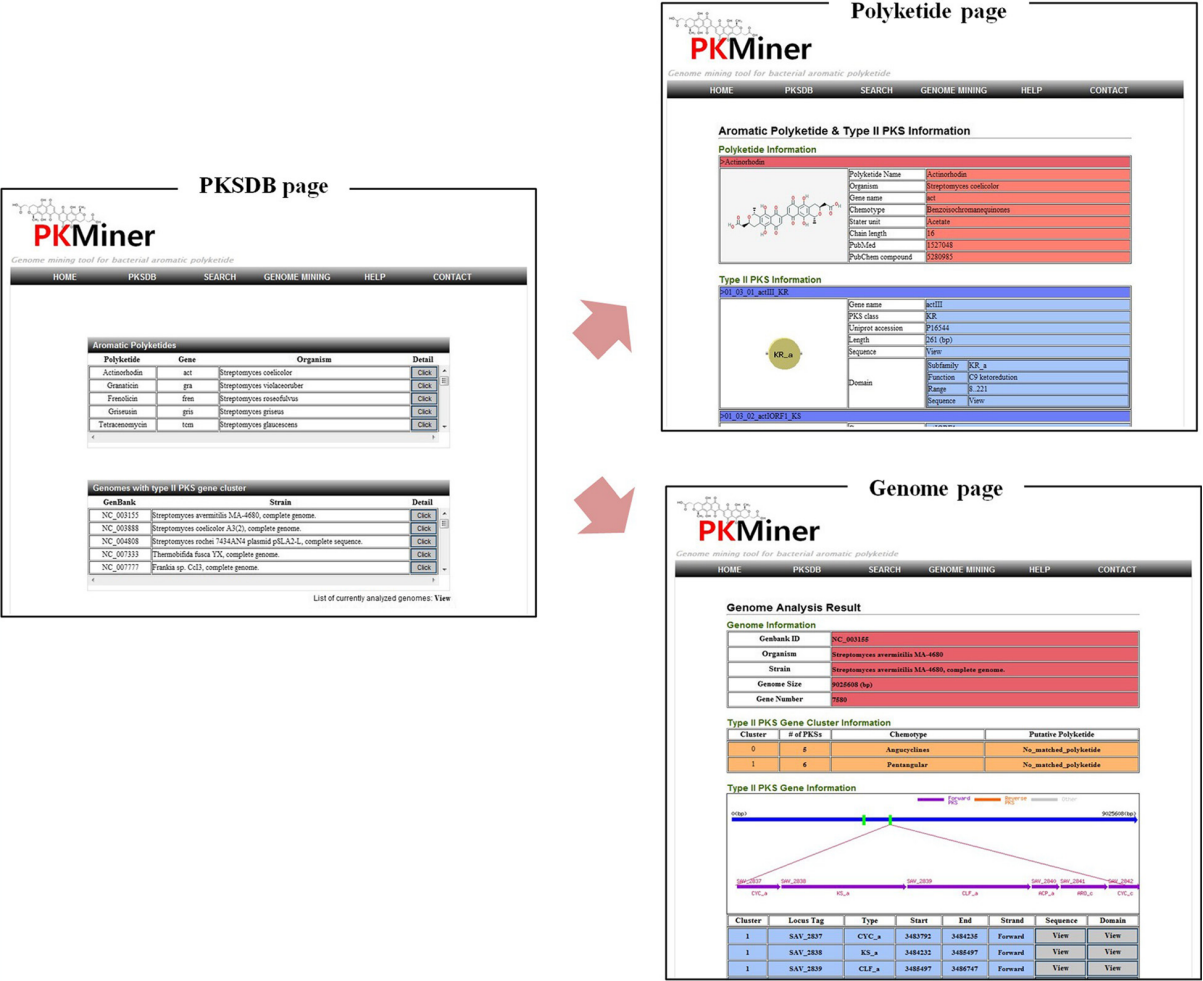
The database interfaces: the browsing page, the polyketide page, and the genome page.

All the results of our analysis were organized into easy-to-use database PKMiner as shown in Figure 2. PKMiner provides known type II PKSs identified from aromatic polyketide gene cluster and predicted type II PKSs resulted from genome analysis. User can explore detail information of aromatic polyketide, type II PKS and the results of genome analysis by clicking the button in detail column. Each entry in polyketide and genome is linked to detail information page of polyketides and genomes

### The search interface

**Figure 3:**
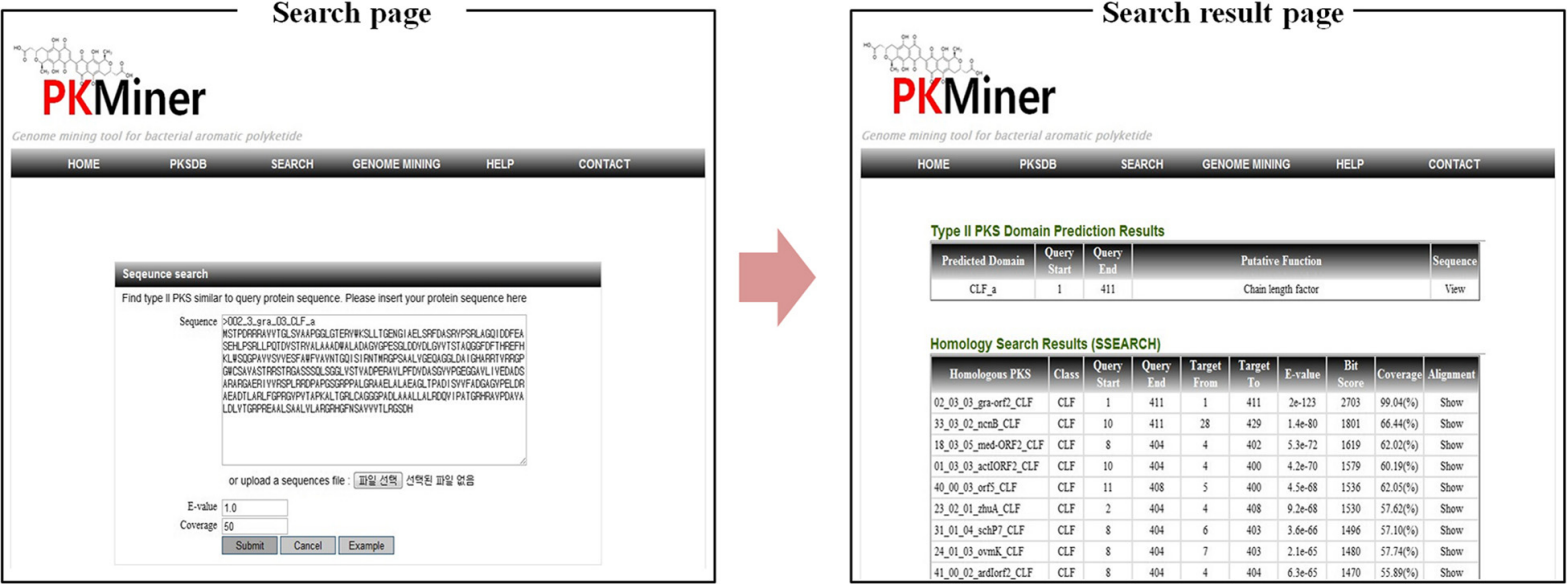
The search interfaces: the search page, and the search result page.

The sequence-based search allows users to quickly find similar type II PKS to the query using type II PKS domain classifiers as shown in Figure 3. User can perform flexible homology search for type II PKS by designating sequence coverage and E-value of SSEARCH. The sequence coverage means the percentage of query sequence alignment to target sequence. The result page shows predicted type II PKS domains and homologs housed in PKMiner.

### The genome mining interface

**Figure 4:**
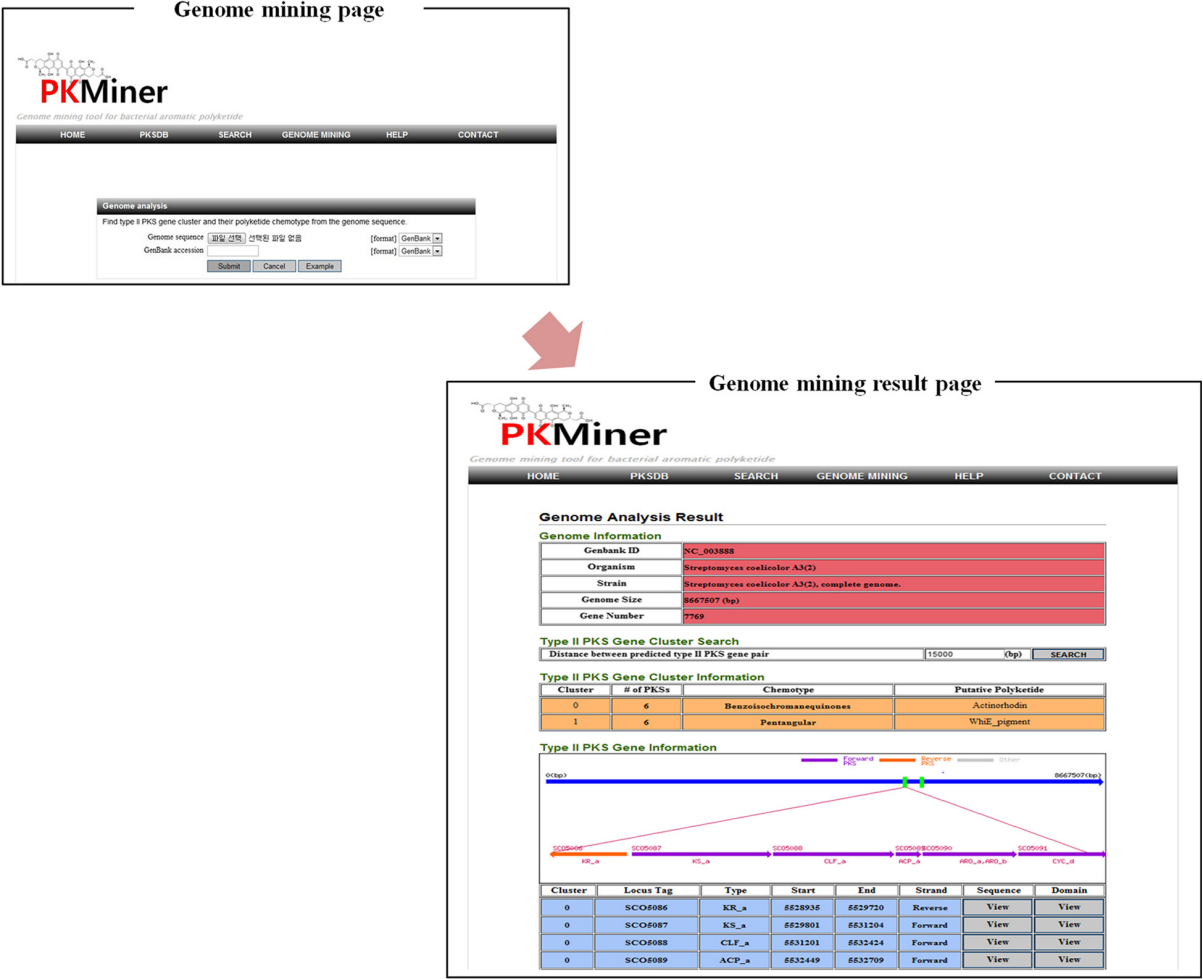
The genome mining interfaces: the genome mining page, and the genome mining result page.

Genome mining interface provides two methods for the analysis of genome sequence. User can upload genome sequence in form of genbank or fasta format. User can also insert genbank accession instead of uploading genome sequence. In case of genome sequence in form of fasta format, PKMiner predict ORF from genome sequence using Glimmer trained with genome sequence of *Steptomyces Coelicolor*. After the analysis of genome sequences, user can examine and manipulate the result of our analysis through interactive analysis tools shown in Figure 4.

## Discussion

We have performed a comprehensive computational analysis of type II PKSs and their gene clusters for the identification of type II PKSs and the prediction of polyketide chemotypes in actinobacterial genomes. Even though subclasses of type II PKS have been inferred from the chemical structure of the aromatic polyketide, earlier studies have not specifically defined subclasses within type II PKS class based on their biosynthetic functions and sequence patterns. We solved this issues using homology based sequence clustering analysis of known type II PKSs. The results of this analysis showed that several type II PKS classes such as KR, ARO, CYC could be separated into type II PKS subclasses with different biosynthetic function. Furthermore, we could identify domain subfamilies of type II PKSs by using sequence patterns of type II PKS subclasses. These results imply that several type II PKS classes could be more sophisticatedly classified into subclasses based on patterns of domain sequences and various different types of aromatic polyketides are synthesized by different biosynthetic pathway catalyzed by type II PKS subclasses.

The identification of type II PKS subclasses enabled us to make prediction rules for aromatic polyketide chemotype corresponding to the combination of type II PKS domains. It has been known that aromatic polyketide is synthesized by various biosynthetic processes including starter unit selection, chain length determination, folding pattern determination, chain tailoring such as methylation, glycosylation and so on. Several previous studies have reported key factors by correlating individual type II PKS sequence with chemical structure of aromatic polyketide [30,31]. Based on previous reports, we tried to deduce general rules applicable to our known type II PKSs for various biosynthetic processes of aromatic polyketide formation. However, we could only find correlation between ARO/CYC domain combination and carbon chain folding pattern for our known type II PKSs.

The development of type II PKS domain classifiers and derivation of prediction rule for aromatic polyketide chemotype allowed us to identify and analyze type II PKS gene cluster. It is important to predict aromatic polyketide chemotype by analyzing type II PKS gene cluster. The aromatic polyketide chemotype provides a framework to understand the type II PKS gene cluster within the known biosynthetic pathway. It also suggests the potential function of individual type II PKS in polyketide biosynthesis pathway. Furthermore, it provides a possibility to design novel aromatic polyketide by engineering the biosynthetic pathway through substitution of type II PKS.

The integration of the type II PKS domain classifiers with the chemotype-prediction rules leaded to development of PKMiner, which can detect type II PKS gene cluster, provides type II PKS functional annotation and predicts the polyketide chemotype of type II PKS product. Compared to previous software antiSMASH, the analysis functionalities described here are unique features in analyzing type II PKS gene cluster. Even though the antiSMASH provides various analysis functionalities such as gene cluster detection, function annotation, prediction of chemical structure, comparative gene cluster analysis and phylogenetic analysis, some of analysis functionalities such as gene cluster detection, comparative gene cluster analysis and phylogenetic analysis are only effective in analyzing type II PKS gene cluster because it lacks comprehensive type II PKS specific domain classifiers and aromatic polyketide structure prediction module.

Genome analysis and literature based validation showed that our method can be successfully applied to identify type II PKSs and predict aromatic polyketide chemotype by analyzing type II PKS gene clusters. Especially, it turns out that pentangular polyphenol is the most abundant polyketide chemotype predicted by the largest number of organisms. However, this approach has potential limitations in type II PKS domain identification and aromatic polyketide prediction. Because our domain classifiers and polyketide chemotype prediction rules always depend on known type II PKS information and type II PKS domain organization, it can miss some totally new types of PKS subclasses or failed to predict aromatic polyketide chemotype with novel domain combination for existing or novel aromatic polyketide chemotype. For example, 9 potential type II PKSs in *Steptomyces avermitilis* MA-4680 were reported based on their general similarity to type II PKSs, but these did not show distinguished sequence similarity to any of our type II PKS domains and their PKS activities have not been validated experimentally [27]. We consider including these type II PKSs into a separate domain subfamily group after their type II PKS activities are proved.

The result of genome analysis remains taxonomic characteristics of microorganisms with type II PSK gene clusters. We thus investigated taxonomic distribution for the above results in more detail. To estimate relative abundance of type II PKS containing genomes between different taxonomic groups, we calculated the ratio between the type II PKS containing genomes and total sequenced genomes in taxonomic hierarchy as a taxonomic group ratio. We chose the suborder as criteria taxon for calculating the taxonomic group ratio because it is known that microorganisms belonging to the order *Actinomycetales* are fascinatingly diverse. Currently, 319 actinobacterial genomes are classified into 6 orders, 17 suborders and 41 families in the NCBI taxonomy. Table 5 shows taxonomic distribution of microorganisms with type II PKS gene clusters. For each of the different suborders, Table 5 shows total number of sequenced genomes, the number of type II PKS containing genomes and the taxonomic group ratio. As can be seen, type II PKS containing genomes exhibited certain taxon-specific distribution. The microorganisms with type II PKS containing genomes are only included in the suborder *Catenulisporineae, Frankineae, Micrococcineae, Micromonosporineae, Pseudonocardineae, Streptosporangineae* and *Streptosporangineae*. Interestingly, the taxonomic PKS group ratio shows that the microorganisms included in suborder *Frankineae, Micromonosporineae, Streptosporangineae* and *Streptosporangineae* have relatively high proportion type II PKS containing genomes, whereas microorganisms included in the suborder *Actinomycineae,Corynebacterineae, Glycomycineae, Kineosporiineae* and *Propionibacterineae* does not have any type II PKS gene clusters. Remarkably, the suborder *Streptosporangineae* which includes genus *Steptomyces* known as prolific taxa for polyketide synthesis is not top rank suborder in taxonomic group ratio. This result suggests that there exist other aromatic polyketide prolific sources besides *Streptosporangineae*.

**Table 5:** Taxonomical distribution of microorganisms with type II PKS gene clusters

**Order**	**Suborder**	**# of sequenced genome**	**# of genomes with type II PKSs**	**Taxonomic PKS group ratio (%)**
*Acidimicrobiales*	*Acidimicrobineae*	1	0	0.00
*Actinomycetales*	*Actinomycineae*	4	0	0.00
*Actinomycetales*	*Catenulisporineae*	1	1	100.00
*Actinomycetales*	*Corynebacterineae*	129	0	0.00
*Actinomycetales*	*Frankineae*	11	6	54.55
*Actinomycetales*	*Glycomycineae*	1	0	0.00
*Actinomycetales*	*Kineosporiineae*	3	0	0.00
*Actinomycetales*	*Micrococcineae*	48	1	2.08
*Actinomycetales*	*Micromonosporineae*	7	5	71.43
*Actinomycetales*	*Propionibacterineae*	12	0	0.00
*Actinomycetales*	*Pseudonocardineae*	11	2	18.18
*Actinomycetales*	*Streptomycineae*	36	6	16.67
*Actinomycetales*	*Streptosporangineae*	7	4	57.14
*Bifidobacteriales*	*Bifidobacteriaceae*	40	0	0.00
*Coriobacteriales*	*Coriobacterineae*	6	0	0.00
*Rubrobacterales*	*Rubrobacterineae*	1	0	0.00
*Solirubrobacterales*	*Conexibacteraceae*	1	0	0.00

For each suborder, this table shows the number of sequence genomes, number of genomes with type II PKSs and taxonomic PKS group ratio. The taxonomic PKS group ratio represents the proportion of the type II PKS containing genomes to total sequenced genomes in the suborder.

## Conclusion

We performed a comprehensive computational analysis of type II PKSs and their gene clusters in actinobacterial genomes. We have developed type II PKS domain classifiers and derived aromatic polyketide chemotype-prediction rules for the analysis of type II PKS gene clusters observed in bacterial genomes. These rules were effective in identifying novel candidates of type II PKS gene clusters and their possible polyketide chemotypes in the available actinobacterial genome sequences. The results of this analysis gave new insights about the distribution of aromatic polyketide chemotypes that can be produced by actinomycetes. This resource can be similarly applied for the analysis of any other known or newly sequenced microorganisms. Furthermore, our tools and the results of this analysis have a potential to be used in microbial engineering to produce various aromatic polyketides by combining the suggested type II PKS modules for the specific aromatic polyketides.

### Availability and requirements

PKMiner is freely accessible for research activity and non-commercial use at the URL: http://pks.kaist.ac.kr/pkminer.

## Abbreviations

PKS, polyketide synthase; KS, keto synthase; CLF, chain length factor; ACP, acyl carrier protein; KR, keto reductase (KR); ARO, aromatase; CYC, cyclase; KSIII, keto synthase III; AL, acyl CoA ligase; AT, acyl transferase; MCAT, malonyl-CoA: ACP transacylase; TE, thioesterase; HMM, hidden Markov Model; SVM, support vector machine; bp, base pair.

## Competing interests

The authors declare that they have no competing interests.

## Authors’ contributions

JK developed methods and analyzed the data. GSY designed and supervised this study. JK and GSY both wrote the manuscript together. All authors read and approved the final manuscript.

## Supplementary Material

Additional file 1 **Table S1. **List of 42 known aromatic polyketide and their gene cluster used for analysis in this study. For each type II PKS gene cluster, this table includes polyketide name, gene name, chemotype, organism, NCBI code and reference. Table S2 List of actinobacterial genomes used for analysis in this study. This table includes NCBI code and species name. Table S3 List of 280 known type II PKSs identified from 42 type II PKS gene clusters. This table includes gene name, protein sequence, protein length, type II PKS class, uniprot accession, Pfam accession and CDD accession. Insignificant hit in Pfam search is given in parenthesis in Pfam column. Table S4 List of 308 type II PKS domains resulted from homology based clustering analysis. This table includes gene name, domain start, end, length, and type. Table S5 List of type II PKS domains in each type II PKS gene cluster for each aromatic polyketide chemotypes. Table S6 List of predicted type II PKSs from the analysis of actinobacterial genomes. This table includes NCBI code, cluster number, protein id, predicted PKS class, homologs, evalue, start, end, direction, locus tag, protein name. Click here for file
